# TGF-β Suppresses β-Catenin-Dependent Tolerogenic Activation Program in Dendritic Cells

**DOI:** 10.1371/journal.pone.0020099

**Published:** 2011-05-20

**Authors:** Bryan Vander Lugt, Zachary T. Beck, Robert C. Fuhlbrigge, Nir Hacohen, James J. Campbell, Marianne Boes

**Affiliations:** 1 Department of Dermatology, Brigham and Women's Hospital, Boston, Massachusetts, United States of America; 2 Center for Immunology and Inflammatory Diseases, Division of Rheumatology, Allergy, and Immunology, Massachusetts General Hospital, Charlestown, Massachusetts, United States of America; 3 Broad Institute of Harvard and MIT, Cambridge, Massachusetts, United States of America; 4 Division of Pediatric Immunology, University Medical Center Utrecht, Wilhelmina Children's Hospital, Utrecht, The Netherlands; Pavillon Kirmisson, France

## Abstract

The mechanisms that underlie the critical dendritic cell (DC) function in maintainance of peripheral immune tolerance are incompletely understood, although the β-catenin signaling pathway is critical for this role. The molecular details by which β-catenin signaling is regulated in DCs are unknown. Mechanical disruption of murine bone marrow-derived DC (BMDC) clusters activates DCs while maintaining their tolerogenic potential and this activation is associated with β-catenin signaling, providing a useful model with which to explore tolerance-associated β-catenin signaling in DCs. In this report, we demonstrate novel molecular features of the signaling events that control DC activation in response to mechanical stimulation. Non-canonical β-catenin signaling is an essential component of this tolerogenic activation and is modulated by adhesion molecules, including integrins. This unique β-catenin-dependent signaling pathway is constitutively active at low levels, suggesting that mechanical stimulation is not necessarily required for induction of this unique activation program. We additionally find that the immunomodulatory cytokine TGF-β antagonizes β-catenin in DCs, thereby selectively suppressing signaling associated with tolerogenic DC activation while having no impact on LPS-induced, β-catenin-independent immunogenic activation. These findings provide new molecular insight into the regulation of a critical signaling pathway for DC function in peripheral immune tolerance.

## Introduction

In contrast to significant advances made towards understanding the signals that control dendritic cell (DC) function in activating T cells during inflammation (immunogenic function), relatively little is known about the signals that control DC function in suppressing inappropriate T cell responses during steady state (tolerogenic function) [1], [2]. Manicassamy *et al.* recently demonstrated that β-catenin signaling is an important component of tolerogenic DC function in peripheral immune tolerance [3]. Mice in which β-catenin signaling is selectively ablated in DCs show striking defects in DC-mediated Treg homeostasis and disease susceptibility. Elucidating the mechanisms by which β-catenin signaling is regulated in DCs may therefore yield insight into the molecular basis for tolerogenic DC function and provide novel targets for therapeutic manipulation of antigen-specific immune responses.

Murine bone marrow-derived DCs (BMDCs) respond to a wide range of stimuli, including sterile mechanical stimulation [4]. Mechanical disruption of cell clusters induces an activation program. that is distinct from the activation induced by TLR ligands such as LPS [5]. Whereas DCs activated by TLR ligands acquire the capacity to stimulate T cell immunity, BMDCs activated by cluster disruption have immunophenotypic characteristics of mature DCs, yet functionally resemble naïve DCs in their ability to promote T cell tolerance. They stimulate T cells to produce cytokine profiles associated with immune tolerance, and will protect against autoimmune disease when used to immunize recipient mice (6). Activation of DCs in this model (hereafter refered to as tolerogenic activation) is associated with β-catenin signaling that is distinct from signaling pathways commonly associated with DC responses to inflammatory stimuli such as TLR ligands [6]. This model is therefore a useful tool to explore the association between β-catenin signaling and tolerogenic activation, as well as to explore how this critical pathway is regulated in DCs.

The balance between immunity and tolerance is regulated at many levels, including by cytokine signals. TGF-β is a cytokine whose signaling is strongly associated with immune suppression and tolerance [7], [8]. TGF-β suppresses DC responses to non-pathogen-associated stimuli [9], suggesting the possibility that TGF-β may counteract non-inflammatory β-catenin activation pathways in DCs. TGF-β signaling is indeed a known regulator of β-catenin signaling [10]. In this report, we have exploited the BMDC model in order to explore the role of β-catenin signaling in tolerogenic DC activation as well as to address to what extent TGF-β influences β-catenin signaling and tolerogenic responses in DCs.

## Methods

### Mice

WT C57BL/6 mice were purchased from the Jackson Laboratories. CD11b^-/-^ mice were generously provided by T. Mayadas (Harvard Medical School). Animal studies were approved by the Brigham and Womens' Hospital/Harvard Medical School institutional review and ethics committee (approval ID of permit number 02726). Mice were maintained in specific pathogen-free conditions in accordance with institutional guidelines.

### Cell culture

Bone marrow-derived dendritic cells were cultured as previously described [11]. In brief, bone marrow was flushed from the femur and tibia, RBC were lysed, and the remaining cells were cultured at 10^6^ cells/ml in RPMI medium with 10 ng/ml murine GM-CSF (Peprotech) and various concentrations of CHO-derived human TGF-β1 (Peprotech). Media was refreshed every other day.

### Cell stimulation and flow cytometry

Inflammatory stimulation: Day 5 BMDCs were stimulated for 24 hours with 100 ng/ml LPS (Sigma-Aldrich). Tolerogenic stimulation: loosely adherent day 5 BMDCs were collected by repeated pipetting and resuspended in fresh media for 24 hours. In some cases, CD11c magnetic beads (Miltenyi Biotec) were used according to manufacturer's instructions to simultaneously purify CD11c+ cells and introduce mechanical stimulation. Integrin-mediated stimulation: LEAF-purified antibodies (Biolegend) were added to cultured cells to 1.5 µg/ml final concentration for 24 hours. Following stimulation, loosely adherent cells were collected by pipetting, for analysis by flow cytometry.

### Calculation of Induced Maturation

To calculate the impact of TGF-β on DC responsiveness to stimulation required that we first correct for the effect of TGF-β on unstimulated levels of spontaneously mature DCs. Percent induced maturation is therefore the percentage of MHC-II^hi^/CD86^+^/CCR7^+^ DCs in the stimulated condition after subtracting the percentage in the unstimulated condition. Fold-induced maturation is calculated as the ratio of induced maturation in the experimental group to the induced maturation in the control group.

### Lentiviral shRNA infection

pLKO vector-encoded lentivirus was produced as previously described [12]. BMDCs were cultured for 2 days with TGF-β before spin infection with 8 ug/ml polybrene at 2 MOI. Medium was replaced at day 4 with fresh medium containing 1 mM TGF-β Receptor Kinase Inhibitor (TRI) (Calbiochem) and 5 uM puromycin (Sigma). For stimulation studies, cells were stimulated 2 days following selection and analyzed 24 hours after stimulation.

### Western blots

Immunoprecipitations were completed using anti-E-Cadherin (DECMA, Abcam) with lysates prepared from CD11c-purified BMDCs using the Pierce Classic IP Kit according to manufacturer's instructions. Protein fractions were run on 4–20% polyacrylamide gels (Biorad) and transferred to PVFD membranes. After blocking, membranes were stained either with unconjugated whole rabbit anti-serum against β-catenin (Sigma) or unconjugated anti-tubulin (Cell Signaling). HRP-conjugated goat anti-rabbit (Cell Signaling) was used as a secondary stain.

### Reporter Assays

Day 2 control or TGF-β BMDCs were infected with lentiviral reporter constructs (Cignal, SABiosciences) following the protocol outlined above with MOI 2. Infected cells were maintained in control or TGF-β medium until analysis on D5. BMDC lysates were prepared and analyzed using a luciferase assay kit (Agilent Technologies) according to manufacturers instructions.

### Epifluorescence Microscopy

BMDCs were cultured for 5 days. CD11c+ cells were purified by magnetic beads (Miltenyi) and plated onto glass coverslips. Cells were allowed to adhere for 24 hours before fixation with 4% paraformaldehyde. Cells were then permeabilized and stained with antibodies or phalloidin (actin), were washed, and mounted with DAPI-containing medium (Invitrogen).

## Results

### BMDCs Recapitulate Tolerogenic versus Imunogenic DC Function

**Figure 1:**
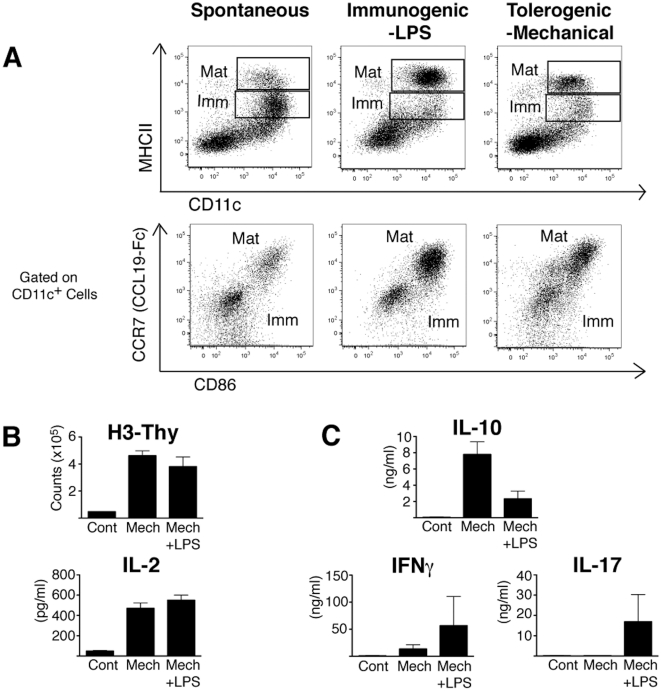
BMDCs Recapitulate Tolerogenic vs Immunogenic DC Function. (A) BMDCs were cultured for 5 days and either left unstimulated (spontaneous maturation, *upper left panel*), treated with LPS (*upper middle panel*), or stimulated by mechanical agitation (*upper right panel*). Twenty four hours after stimulation, BMDCs were collected and analyzed by flow cytometry for maturation status. Mature (Mat) BMDCs were identified as CD11c^+^MHCII^hi^CD86^hi^CCR7^hi^. Immature (Imm) are CD11c^+^MHCII^int^CD86^-^CCR7^-^. (B,C) Day 5 BMDCs were purified/mechanically-stimulated with CD11c magnetic beads and replated with 100 ng/ml LPS (Mech+LPS) or without (Mech). Stimulated cells were pulsed for 2 hours with chicken ovalbumin peptide OVA 323–339 (10 µg/ml) and washed before injection i.v. into recipient mice. Recipient mice received treated DC injections on days 0,2, and 4. On day 7, splenocytes from recipient mice and control mice that had received no injections (Cont) were harvested and rechallenged *ex vivo* with OVA peptide for 72 hours. (B) Proliferation was measured either by adding H^3^Thy during the final 18 hours of challenge or by ELISA for IL-2 levels in the culture supernatant. (C) Cytokine levels in culture supernatant associated with tolerance (IL-10) and immunity (IFNγ and IL-17) were determined by ELISA. *p<0.05. FACS plots in part A are single experiments representative of more than 3 repeats. Graphs in B,C show data from 3 experiments as mean+/−SEM.

We cultured immature bone marrow-derived dendritic cells (BMDCs) to study mechanisms that may orchestrate tolerogenic DC maturation. We began our studies by reproducing aspects of this model reported previously (Figure 1). DC maturation encompasses many changes in protein expression and function, including alterations in the transcript levels of hundreds of genes, with different stimuli resulting in different patterns of transcriptional responses [13]. As current nomenclature is inadequate to describe variable DC activation states, for the purposes of this report we will define a mature DC as an activated cell that displays enhanced antigen presentation capability. At a minimum, this requires increased surface display of MHCII-peptide complexes and costimulatory molecules (i.e. CD86), as well as expression of the chemokine receptor CCR7 that allows migrating DCs to reach tissue-draining lymph nodes and interact with T cells therein. For simplicity, we will therefore define a mature DC as one expressing high levels of these three molecules.

As defined by our criteria, a relatively small percentage of BMDCs underwent spontaneous maturation without manipulation (identified by the MHCII/CD86/CCR7^hi^ immunophenotype) (Figure 1A). We find that levels of spontaneous maturation range from 5–20% in our hands. However, maturation was dramatically increased above spontaneous levels upon exposure to inflammatory stimulation (*i.e.*, LPS). As previously reported, we also find that maturation is robustly induced by mechanical disruption of BMDC clusters (*i.e.* stimulation by mechanical agitation). We confirmed by flow cytometry that both types of maturation stimuli activated the “core” aspects of the DC maturation response (Figure 1A).

BMDCs induced to mature by exposure to LPS are reported to orchestrate immunogenic T cell responses, while those matured by mechanical stimulation can coordinate tolerogenic responses [6]. We confirmed this important distinction with a functional test similar to that first described with this model [6]. We stimulated BMDC cultures either with LPS or with mechanical stimulation, pulsed the activated DCs with antigen, and used the DCs to immunize recipient mice. After 3 immunizations we harvested spleens from recipient mice (using spleens from mice that had received no immunizations as controls) and challenged splenocytes *ex vivo* with cognate antigen. We find that while both LPS- and mechanically-stimulated BMDCs prime the recall response equally well (as measured by ^3^H-thymidine uptake and IL-2 production [Figure 1B]), the cytokine profile elicited by mechanically-stimulated BMDCs (high IL-10, low IFN-γ, low IL-17) was distinct from that induced by LPS-stimulated BMDCs and consistent with immune tolerance (Figure 1C).

### Direct Perturbation of Integrins Mimics Intrinsic BMDC Response to Mechanical Signals

As an initial step to understanding tolerogenic function in BMDCs, we sought to better understand the molecular mechanisms by which mechanical stimulation induces maturation. Previous studies with mechanical stimulation have utilized relatively poorly defined stimuli to introduce mechanical stimulation, such as simple pipetting [4] or purification with magnetic beads [6], which has hampered the ability to precisely identify the molecular mechanisms that mediate the ensuing response. It has been proposed that mechanical disruption of homotypic E-Cadherin interactions between adjacent BMDCs initiates β-catenin signaling and the tolerogenic response [6]. We found, however, that individual BMDCs can respond to mechanical signals independently of the disruption of cell-cell interactions (Figure S1). Therefore, BMDCs appear intrinsically capable of responding to mechanical signals. This finding implicates the involvement of alternative molecules in addition to E-Cadherin in the response to mechanical stimulation.

**Figure 2:**
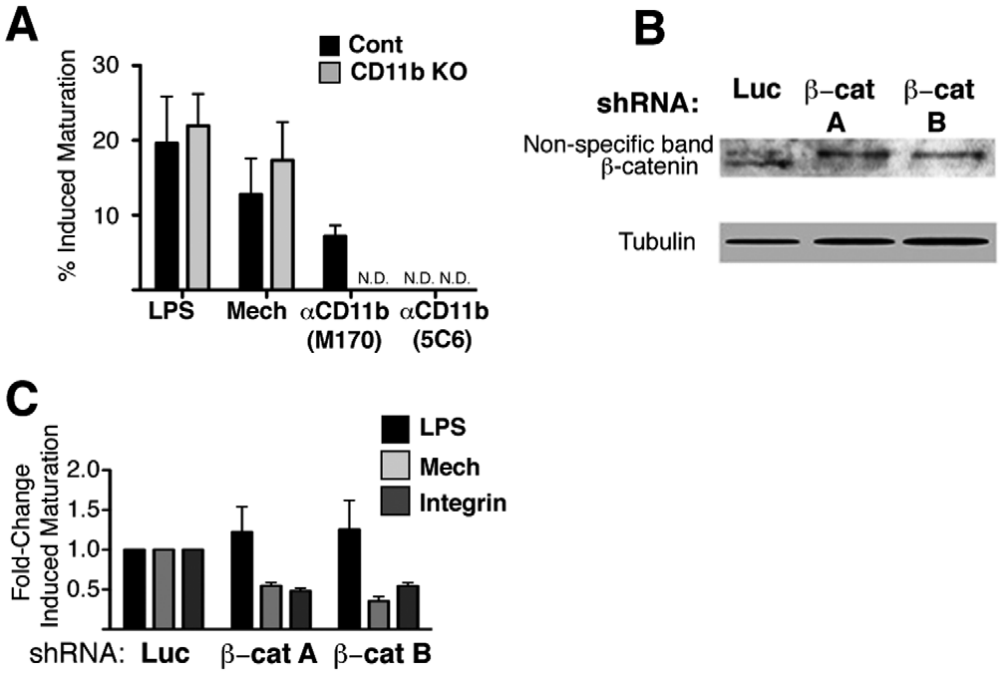
Tolerogenic Response to Mechanical Stimulation is Controlled by a Distinct Signaling Pathway. (A) Day 5 WT or CD11b^-/-^ BMDCs were stimulated with 100 ng/ml LPS, with mechanical agitation, or by adding 1.5 ug/ml anti-CD11b (clones M170 or 5C6) to culture medium. 24 hours later maturation status was assessed by flow cytometry as in Fig 1. To correct for TGF-β-mediated differences in levels of spontaneous maturation, we calculate percent “induced maturation” as described in the methods section. The percentage of spontaneously mature DCs was comparable between WT and CD11b^-/-^.DCs Data from 3 experiments is summarized as mean ± SEM. N.D. Not Detected. (B) BMDCs were infected with 2 independent lentiviral shRNA clones against β-catenin, or with control shRNA against luciferase. Cell extracts were prepared 6 days after infection and knockdown efficiency was confirmed by western blot. Blots are representative of 3 experiments. (C) BMDCs were infected as in (B). Infected BMDCs were stimulated with LPS, mechanical agitation, or by integrin stimulation (αCD11b clone M170) and analyzed 24 hours later by flow cytometry. Data from 3 experiments is summarized as mean +/- SEM.

In addition to cadherins, integrins also make important contributions to cellular responses to mechanical signals [14], [15]. DCs express high levels of β2 integrins, including CD11b (αM) and the DC lineage-associated CD11c (αX). We reasoned that if integrins are involved in initiating signaling events in response to mechanical stimulation, it might be possible to mimic mechanical stimulation with a defined stimulus against BMDC integrins. This approach also has the desirable advantage of circumventing the poorly defined stimulus of repeated pipetting to introduce mechanical signals. To determine whether direct stimulation of integrins initiates maturation similar to that induced by mechanical agitation, we stimulated BMDC cultures with an antibody to CD11b. We found that integrins can indeed initiate maturation, albeit to slightly lower levels than that observed with mechanical stimulation (Figure 2A). This response to CD11b MAb was dependent on the presence of CD11b, as evidenced by the absence of a similar response from CD11b^-/-^ BMDCs (Figure 2A). However, the BMDC response to mechanical stimulation did not require the participation of CD11b under our standard conditions, as it occurred normally in cells from CD11b^-/-^ mice (Figure 2A). We conclude that although integrins such as CD11b can facilitate DC maturation consistent with mechanical agitation, the tolerogenic response to mechanical stimulation is not uniquely dependent on a single integrin.

Interestingly, we found that the ability of anti-CD11b antibodies to initiate integrin-mediated activation varied by clone. While clones M170 and 5C6 bind with equal efficiency to CD11b (Figure S2), clone M170 induced maturation but clone 5C6 did not (Figure 2A). This suggests that integrin crosslinking alone is not sufficient to initiate maturation signals. The binding sites for these two antibodies have not been mapped, but are evidently not in close enough proximity to block each other's binding (Figure S2). We propose that the antibody-induced stimulatory effect on DCs depends upon the MAb's ability either to stabilize or force an activated integrin conformation. We also observe that the addition of the M170 clone (but not other clones) to cultured BMDCs results in the CD11b-dependent formation of large clusters of cells within 1 hour, further suggesting that disruption of cells clusters is not a critical component of the response to mechanical signals (BJV, data not shown).

### β-catenin Signaling is Necessary for Tolerogenic Mechanical Response and Spontaneous Maturation

β-catenin signaling is an important component of tolerogenic DC function *in vivo*. Consistent with this observation, mechanical stimulation, which activates DCs while preserving their tolerogenic potential, is known to induce β-catenin signaling in BMDCs. Experimentally-induced β-catenin signaling is *sufficient* to promote DC maturation [6]; however, it remains unclear whether β-catenin signaling is *necessary* for the generation of tolerogenic DCs by mechanical stimulation.

To determine whether mechanical stimulation and/or integrin-mediated activation require β-catenin-mediated signals, we used a lentivirus shRNA knockdown approach to reduce the availability of functional β-catenin. We characterized two independent shRNA clones against β-catenin, each producing a substantial reduction of total β-catenin protein (Figure 2B). BMDCs infected with these shRNAs developed normally, expressed normal levels of CD11c, and remained viable (as measured by scatter profile, Figure S3). While β-catenin-depleted BMDCs responded normally to LPS stimulation, they did not respond to mechanical or integrin-mediated stimulation (Figure 2C). This demonstrates that β-catenin signaling is a necessary component of the activation program induced by mechanical signals.

**Figure 3:**
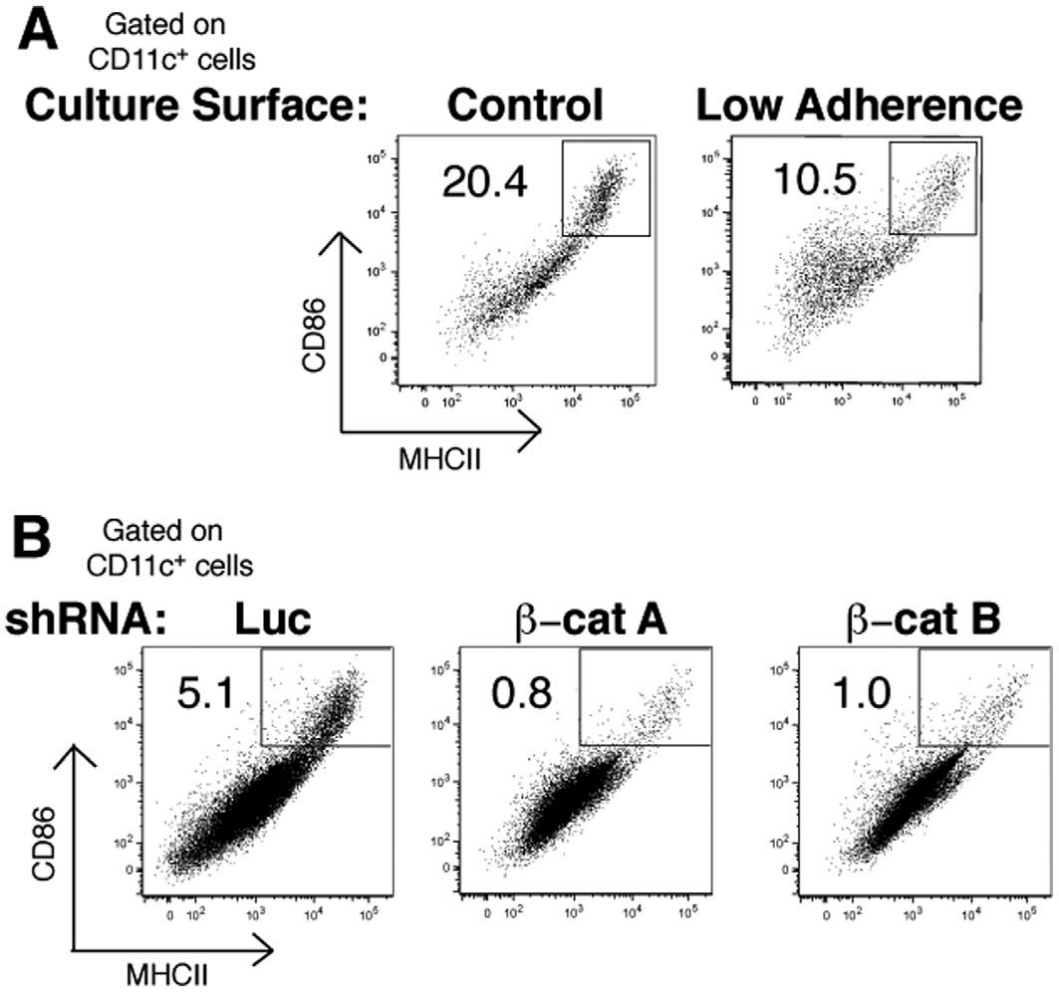
Signals Associated with Tolerogenic Function are Constitutively Active. (A) BMDCs were cultured for 5 days on standard tissue culture treated plates or on low adherence plates (hydrogel-coated, Invitrogen) and then analyzed for maturation status by flow cytometry. Gates are drawn on CD11c^+^MHCII^hi^/CD86^hi^ mature cells. (B) BMDCs were infected with luciferase or β-catenin shRNA as in Fig 2 and cultured for 7 days post-infection before maturation status was assessed by flow cytometry. Mature cells are identified as in (A). Numbers indicate percent within mature gate. Data from parts A&B are representative of at least 3 repeats.

β-catenin signaling is constitutively active in steady state tolerogenic DCs *in vivo*, though it remains unclear whether this activation is initiated by canonical Wnt signals or via alternative means [3]. We therefore investigated whether β-catenin-dependent signals are constitutively active in BMDCs and whether these signals regulate the observed spontaneous BMDC maturation, as they regulate DC maturation induced by mechanical stimulation. To this end, we first cultured BMDCs on low adherence tissue culture surfaces to reduce interactions between cell adhesion molecules and the culture substrate. Although “immature” CD11c^+^ DCs developed normally from BM precursors under these conditions, spontaneous maturation (as measured by upregulation of MHCII and CD86) was reduced with respect to maturation achieved on standard culture substrates (Figure 3A).

We next infected BMDCs with shRNA lentiviruses targeting β-catenin as above, and then cultured the infected cells for an additional 7 days. Although we observe lower levels of spontaneous maturation with our infection protocols (compare leftmost panels of Figure 3A with Figure 3B), we repeatedly observed approximately 80% reduction in the levels of spontaneous maturation in β-catenin-depleted DCs in comparison to control-infected cells (Figure 3B). We conclude that β-catenin-mediated signaling is constitutively active in BMDCs and this signal drives spontaneous maturation.

### TGF-β Selectively Suppresses Spontaneous and Mechanically-stimulated DC Maturation

**Figure 4:**
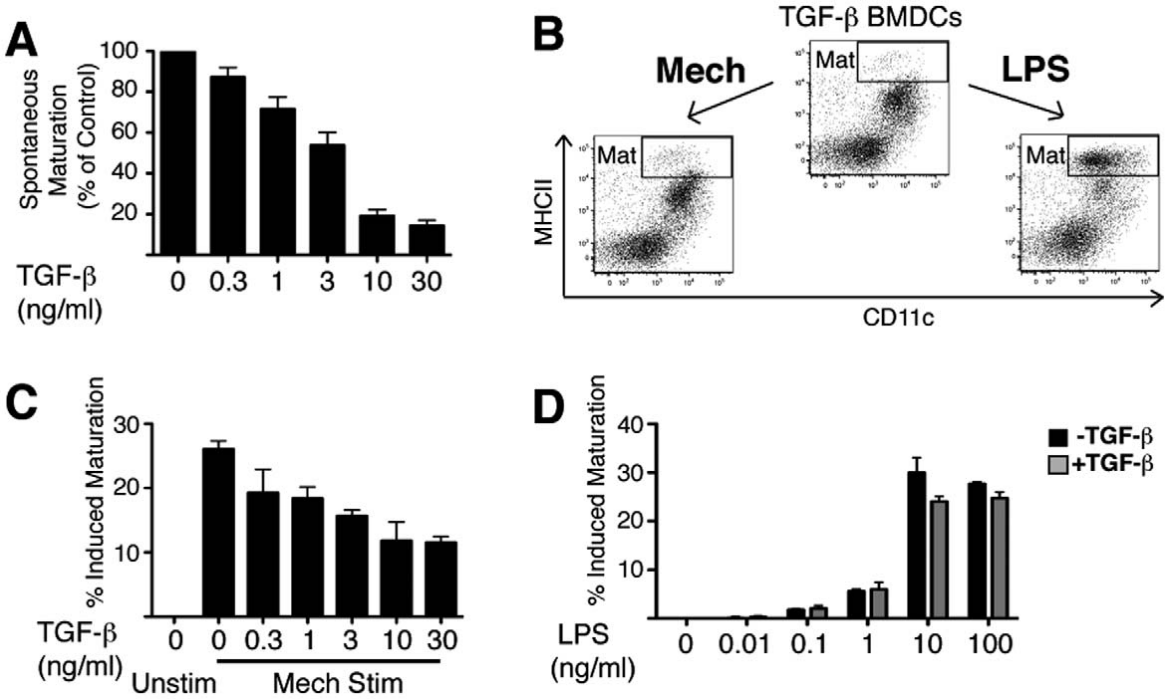
TGF-β Selectively Suppresses Spontaneous and Mechanically-stimulated Maturation. (A) BMDCs were cultured for 5 days in the presence of the indicated TGF-β concentration before analysis for maturation status by flow cytometry as in Fig 1. Graphs indicate levels of spontaneous maturation relative to control cultures. (B) BMDCs cultured in the presence of TGF-β for 5 days were stimulated with either LPS or mechanical agitation before analysis by flow cytometry. Gates are drawn for the CD11c^+^MHCII^hi^ mature (Mat) population. (C) Day 4 control BMDCs were exposed for 24 hours to the indicated concentration of TGF-β, then cells were stimulated by mechanical agitation. After another 24 hours, stimulated cells were collected and analyzed for maturation status by flow cytometry. (D) Cells were cultured as in (C) with TGF-β added to 10 ng/ml. Twenty four hours after the addition of TGF-β, cells were stimulated with the indicated concentration of LPS. After another 24 hours, cells were collected and analyzed for maturation status by flow cytometry. Panels in B are representative examples of at least 3 repeats. Graphs in A,C&D depict the mean+/− SEM of 3 experiments.

TGF-β is a critical regulator both of immune tolerance and of DC biology. TGF-β is also a known regulator of β-catenin signaling. We therefore considered the notion that TGF-β may regulate β-catenin-dependent signaling in DCs. To assess the impact of TGF-β on BMDC maturation, we first cultured BMDCs with titrated concentrations of TGF-β. We found that increasing TGF-β doses correlated inversely with the appearance of spontaneously matured BMDCs (Figure 4A). To determine whether TGF-β rendered BMDCs terminally incapable of maturation, we exposed TGF-β-cultured DCs to either LPS or mechanical stimulation. TGF-β-treated DCs responded robustly to LPS stimulation, indicating that the general maturation program was intact. In contrast, mechanically-stimulated maturation was dramatically inhibited (Figure 4B).

Although LPS-responsive CD11c^+^ “immature” DCs expand from BM at normal rates in the presence of TGF-β (Figure 4B), we considered the possibility that these cells might have been functionally altered with respect to immature DCs that were never exposed to TGF-β. The observed suppression of maturation might be a direct, reversible effect of TGF-β; or an indirect effect resulting from induction of an alternative developmental pathway. To distinguish between these two possibilities, we performed the initial BMDC culture in the absence of exogenous TGF-β, but then added TGF-β for a 24 h period prior to mechanical or LPS stimulation. Under these conditions, TGF-β again suppressed mechanically-stimulated maturation in a dose-dependent manner (Figure 4C), but did not effect LPS-induced maturation at any of the concentrations tested (Figure 4D). These data favor the conclusion that exogenous TGF-β exerts a direct suppressive effect on mechanically-stimulated DC maturation.

### TGF-β Alters BMDC Morphology

**Figure 5:**
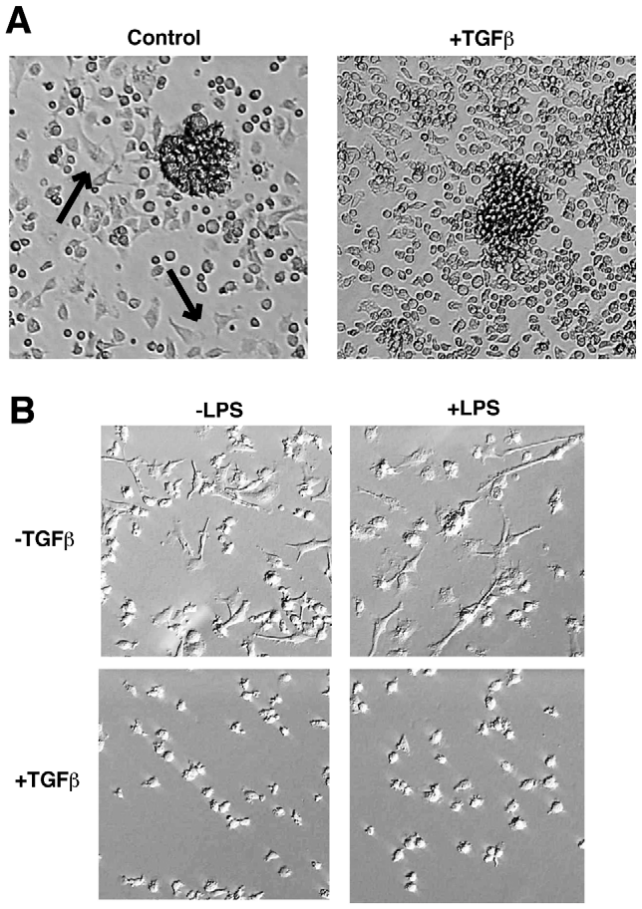
TGF-β Directly Alters DC Morphology. (A) Day 5 BMDCs cultured in the presence or absence of TGF-β were visualized for both cell cluster formation and cell morphology by microscopy. Arrows indicate cells that have spread onto the tissue culture surface. These cells are not apparent in TGF-β cultures. (B) CD11c+ cells cultured as in (A) were purified/mechanically-stimulated with magnetic beads and re-plated in medium with or without 100 ng/ml LPS. Twenty four hours after stimulation cells were analyzed by microscopy. Note the cytoskeletal activation and cell spreading in control DCs that is absent in TGF-β DCs, even when stimulated by LPS. White arrows in upper panels indicate punctate actin staining. All images are representative of at least 3 independent experiments.

Selective suppression of mechanically-stimulated DC maturation by TGF-β indicates that activation by this stimulus is uniquely dependent on a TGF-β-sensitive signaling molecule. We first investigated the impact of TGF-β on DC morphology during culture. We found no effect of TGF-β on BMDC cluster formation (Figure 5A). However, individual cells that were not congregated in cell clusters were noticeably altered in morphology. Without TGF-β, many of these cells displayed a “spread” conformation on the tissue culture surface (Figure 5A, *arrows*). Cytoskeletal activation is associated with DC maturation [16], so these “spread” cells are likely to comprise the spontaneously matured BMDC population observed by flow cytometry (as seen in Figure 1A). Such “spread” cells were not evident in TGF-β-treated DC cultures, where cells that were not congregated in clusters remained comparatively round (Figure 5A). The relative absence of spread cells might simply reflect the observed TGF-β-mediated reduction in spontaneous DC maturation (as seen in Figure 4A). More interestingly however, the decreased cell spreading might instead result from a direct effect of TGF-β on DC cytoskeletal organization or adhesion.

To distinguish these two possibilities, we used magnetic beads to purify CD11c^+^ cells from TGF-β^+^ or TGF-β^-^ BMDC cultures, a process that in itself provides mechanical stimulation [6]. By purifying DC we were also able to confirm that our observations in heterogeneous BMDC cultures were specific to DCs rather than residual macrophages. We re-plated DCs in the presence or absence of LPS for 24 h, and analyzed them for morphological changes by microscopy. We found that TGF-β-treated BMDCs did not spread on the culture surface, regardless of the presence or absence of LPS (Figure 5B). As seen for experiments described above (Figure 4B), the ability of LPS to stimulate core maturation was not inhibited by TGF-β. Thus, TGF-β appears to directly alter DC morphology independently of LPS-inducible “core” maturation.

### TGF-β Disrupts β-catenin Signaling

**Figure 6:**
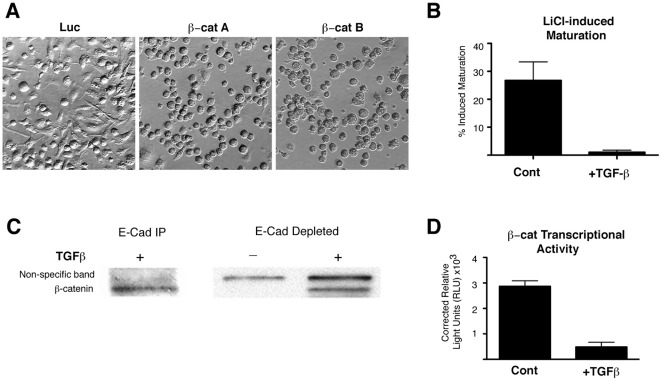
TGF-β Disrupts β-catenin Function. (A) BMDCs were infected with either control or β-catenin shRNA as in Fig 2. shRNA infected cells were analyzed by microscopy 6 days after infection. Images are representative of 3 experiments. (B) BMDCs were cultured for 5 days with or without TGF-β, then stimulated with 10 mM LiCl. 24 hours after stimulation, maturation was assessed by flow cytometry as in Fig 1. Graphs display data from 3 experiments as mean±SEM. (C) BMDCs were cultured for 5 days in the presence (+TGF-β) or absence (Cont) of TGF-β. CD11c^+^ cells were purified on ice with magnetic beads and lysed immediately to avoid mechanical maturation induced changes. E-Cadherin (E-Cad) was immunoprecipitated from lysates and both precipitate and E-Cad depleted fractions were analyzed for β-catenin by western blot. Blots are representative of 2 experiments. (D) Day 2 BMDCs cultured with (+TGF-β) or without (Cont) TGF-β were infected with TCF/LEF lentiviral luciferase reporters. On Day 5, cell lysates were prepared from infected cells, luciferase substrate was added to lysates, and the resulting luminescence was measured. Graph shows data from 3 experiments as mean+/− SEM.

Three observations suggested to us that in addition to the disruption of the cytoskeleton and adhesion molecules, TGF-β additionally disrupts downstream β-catenin signaling. First, although TGF-β reduced the surface levels of CD11b, a substantial amount did remain (Figure S2), yet was not capable of transmitting maturation signals (Figure 2). Second, we noted that β-catenin-depleted BMDCs exhibited a rounded morphology when compared with control infected BMDCs (Figure 6A), consistent with our observations of TGF-β-treated BMDCs (Figure 5B). Third, while treatment of control BMDCs with LiCl (a potent inducer of β-catenin signaling [17]) resulted in maturation, TGF-β-treated BMDCs failed to respond to LiCl (Figure 6B). We therefore reasoned that β-catenin might comprise a direct target of TGF-β-mediated inhibition.

To address this possibility, we examined the effects of TGF-β on β-catenin function. Given the morphological changes we observed after TGF-β treatment, we first examined the structural function of β-catenin. To investigate the possibility that TGF-β disrupts β-catenin/E-Cadherin associations, we immunoprecipitated (IP) E-Cadherin from TGF-β-cultured DC lysates. Western blots demonstrate that TGF-β did not inhibit co-precipitation of β-catenin with E-Cadherin, suggesting that this particular association is not disrupted (Figure 6C). Interestingly, we found that the amount of β-catenin not associated with E-Cadherin was increased in TGF-β-treated cells (Figure 6C).

In addition to its structural role, β-catenin serves as a transcription factor. To evaluate the impact of TGF-β on β-catenin transcriptional activity, we infected BMDCs with a lentiviral TCF/LEF-luciferase reporter. We found that TGF-β suppresses β-catenin transcriptional activity (Figure 6E). Thus, we demonstrate that TGF-β directly regulates β-catenin signaling in DCs, thereby suppressing a critical component required for initiating the unique tolerogenic activation program by mechanical stimulation.

## Discussion

We set out to better define the molecular mechanisms by which mechanical stimulation induces the unique tolerogenic activation program in BMDCs and to investigate the function and regulation of β-catenin signaling in this response. By this approach, we describe several novel features of β-catenin signaling in DCs. Although it has been proposed that disruption of E-Cadherin-mediated cell-cell initiates the tolerogenic program [6], we find that individual BMDCs respond to mechanical stimulation in the absence of cell-cell contacts and that additional adhesion molecules, including integrins, may play an important role in regulating DC activation. We further demonstrate that activated β-catenin is not only sufficient to promote BMDC maturation, it is indeed a necessary component for tolerogenic DC activation by mechanical stimulation. By contrast, LPS-induced immunogenic maturation is independent of β-catenin. Finally, we demonstrate that TGF-β disrupts β-catenin signaling, thereby selectively suppressing this response while leaving immunogenic activation undisturbed.

β-catenin signaling is constitutively active in tolerogenic DC subsets *in vivo* [3], yet the signal that initiates β-catenin signaling in steady state DCs is unknown. Although peripheral tissues like the skin are subject to mechanical agitation, evidence has been lacking as to whether such stimulation may itself be required for steady state DC function [18]. Wnt ligands are expressed by DCs *in vivo* suggesting the possibility of autocrine activation in the absence of external signals [3]. Therefore, it is noteworthy that in the BMDC model, β-catenin-dependent maturation will occur spontaneously in the absence of any exogenous stimulus. We find, however, that adhesive interactions between DCs and the tissue culture surface modulate the extent of spontaneous maturation, indicating that non-canonical signals contribute to the regulation of the β-catenin pathway in DCs. Based on our interpretation of the *in vitro* model, we propose that β-catenin-depdendent tolerogenic DC function *in vivo* may not be driven by mechanical stimulation *per se*, but rather by a constitutively or stochastically active signal modulated by adhesion molecules and intensified by mechanical stimulation. Further studies will be required to determine the contributions of canonical wnt signaling and alternative signaling mediated by adhesion molecules to steady state tolerogenic DC function *in vivo*.

Our data provide insight into mechanisms that may regulate signaling pathways associated with tolerogenic DC function *in vivo*. TGF-β is a well-established regulator of DC development, chemoattraction and function, particularly for DC subsets in peripheral tissues such as skin, the lungs, and the gut [8], [19], [20], [21]. As TGF-**β** is strongly associated with immune suppression, it is perhaps counterintuitive that TGF'β antagonizes a signaling pathway associated with tolerogenic DC function. However, peripheral tissues are continuously subject to mechanical stress that would be predicted to initiate wide-spread activation of DCs. We speculate that TGF-β may act directly to suppress DC activation by opposing β-catenin-dependent signaling. Consistent with this model, mice in which DCs are deficient in TGF-β signaling display enhanced spontaneous DC maturation and migration [22]. It is noteworthy that even at the highest concentrations of TGF-β tested in our *in vitro* model, we continued to observe low levels of spontaneous maturation. Therefore, even in a TGF-β-rich environment our model predicts infrequent but measurable maturation to occur, consistent with *in vivo* observations [23]. As immunogenic activation signaling pathways are not sensitive to TGF-β suppression, robust maturation is predicted upon DC exposure to inflammatory signals. It is also important to note that TGF-β suppresses an activation program that preserves the tolerogenic function found in unstimulated DCs, but would not be expected to inhibit tolerogenic function *per se*.

The unique role of β-catenin in tolerogenic DC function, and the finding that this molecule may be regulated independently of immunogenic DC signaling, is particularly intriguing with respect to therapeutic manipulation of antigen-specific immune responses. It suggests that signaling pathway components uniquely associated with immunogenic or tolerogenic DC function *in vivo* might be selectively targeted, in order that one might “steer” DC function towards a desired functional outcome and thereby shift the balance of immunity and tolerance in human disease. Further research will be required to explore the validity of this concept.

## Supporting Information

Figure S1 **Disruption of cell-cell contacts is not essential for BMDC response to mechanical stimulation.** Day 5 CD11c^+^ BMDCs were purified by magnetic bead separation (Miltenyi). TGF-β was included in initial culture to prevent maturation during purification. Purified DCs were replated at sufficiently low density to minimize cell-cell contact formation. 24 hours after replating, DCs were either left untreated or mechanically-stimulated by repeated pipetting and replated. 48 hours after initial purification, DCs were assessed for maturation by flow cytometry. Plots are representative of 3 experiments.
(TIF)

Figure S2 **Integrins and DC activation.** A) CD11b expression on Day 5 BMDCs was assessed by flow cytometry using either the M170 or 5C6 clones. Grey indicates isotype control staining. Both clones show similar levels of binding. B) BMDCs were either left untreated (Cont) or pretreated with unconjugated 5C6 before staining with PE-conjugated M170. Pretreatment with 5C6 did not block M170 binding. C) CD11a and CD11b expression levels were analyzed by flow cytometry on Day 5 conventional (Cont) or TGF-β-cultured BMDCs.
(TIF)

Figure S3 **Lentiviral shRNA knockdowns in BMDCs.** A) shRNA-infected BMDCs display normal flow cytometry scatter profile after puromycin selection. B) BMDCs infected with control shRNA were analyzed for maturation before and after stimulation by flow cytometry. Stimulated DCs display over 20-fold increase in levels of DCs bearing markers of maturation after stimulation.
(TIF)
