# Supplementary figures and images for: TGF-β Suppresses β-Catenin-Dependent Tolerogenic Activation Program in Dendritic Cells
Source: PLoS One. 2011 May 20;6(5):e20099. doi: 10.1371/journal.pone.0020099 (PMC3098869; doi:10.1371/journal.pone.0020099)

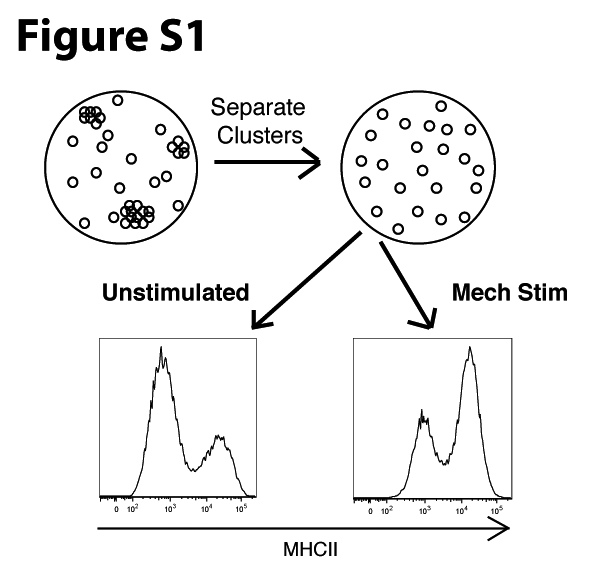

Supplement: Figure S1 — Disruption of cell-cell contacts is not essential for BMDC response to mechanical stimulation. Day 5 CD11c+ BMDCs were purified by magnetic bead separation (Miltenyi). TGF-β was included in initial culture to prevent maturation during purification. Purified DCs were replated at sufficiently low density to minimize cell-cell contact formation. 24 hours after replating, DCs were either left untreated or mechanically-stimulated by repeated pipetting and replated. 48 hours after initial purification, DCs were assessed for maturation by flow cytometry. Plots are representative of 3 experiments. (TIF) [file pone.0020099.s001.tif]

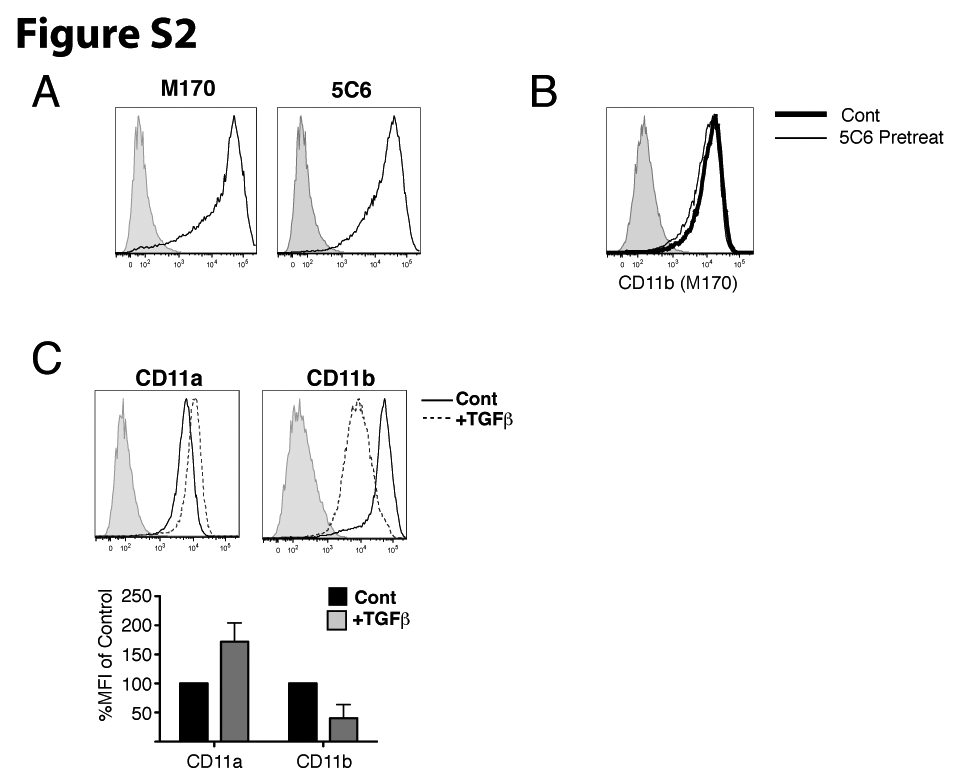

Supplement: Figure S2 — Integrins and DC activation. A) CD11b expression on Day 5 BMDCs was assessed by flow cytometry using either the M170 or 5C6 clones. Grey indicates isotype control staining. Both clones show similar levels of binding. B) BMDCs were either left untreated (Cont) or pretreated with unconjugated 5C6 before staining with PE-conjugated M170. Pretreatment with 5C6 did not block M170 binding. C) CD11a and CD11b expression levels were analyzed by flow cytometry on Day 5 conventional (Cont) or TGF-β-cultured BMDCs. (TIF) [file pone.0020099.s002.tif]

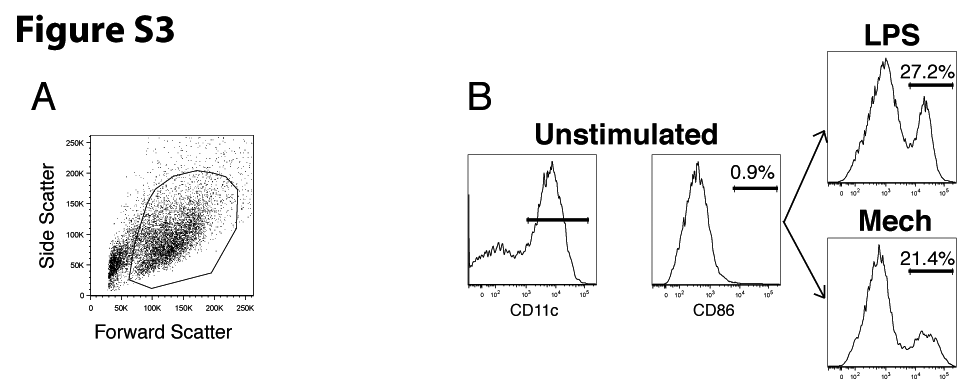

Supplement: Figure S3 — Lentiviral shRNA knockdowns in BMDCs. A) shRNA-infected BMDCs display normal flow cytometry scatter profile after puromycin selection. B) BMDCs infected with control shRNA were analyzed for maturation before and after stimulation by flow cytometry. Stimulated DCs display over 20-fold increase in levels of DCs bearing markers of maturation after stimulation. (TIF) [file pone.0020099.s003.tif]
